# First person – Samanta Sarti

**DOI:** 10.1242/bio.057182

**Published:** 2020-11-06

## Abstract

First Person is a series of interviews with the first authors of a selection of papers published in Biology Open, helping early-career researchers promote themselves alongside their papers. Samanta Sarti is first author on ‘Inducible modulation of miR-204 levels in a zebrafish melanoma model’, published in BiO. Samanta conducted the research described in this article while a PhD student in Laura Poliseno's lab at the Oncogenomics Unit Core Research Laboratory (CRL), ISPRO/Institute of Clinical Physiology (IFC), CNR, in Pisa, Italy. She is now a Postdoctoral researcher in the lab of Samuel Sidi in the Department of Medicine, Division of Hematology and Medical Oncology and Department of Cell, Developmental and Regenerative Biology, at the Tisch Cancer Institute, Icahn School of Medicine at Mount Sinai in New York, investigating the role of miR-204 in melanoma development by its inducible modulation in a zebrafish melanoma model.


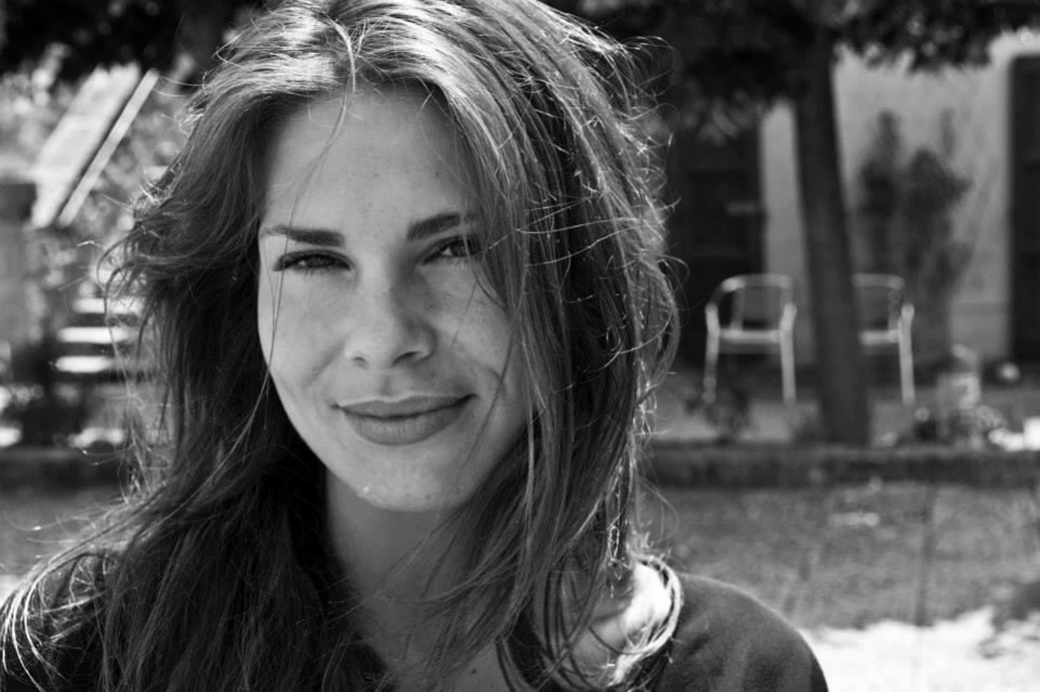


**Samanta Sarti**

**What is your scientific background and the general focus of your lab?**

I have a medical-diagnostic biotechnology background and scientific research has always been my passion. Five years ago, I started my PhD in Laura Poliseno's laboratory at IFC-CNR/ISPRO, where I had the opportunity to join different projects. The main focus of the lab is BRAFV600E oncoprotein and resistance to BRAF inhibitors: we aimed at elucidating the mechanisms at the basis of the functioning and the regulation of BRAFV600E, with particular interest in non-coding RNAs and pigmentation. The ultimate goal is to find reliable therapies to overcome acquired resistance in melanoma patients, improving their prognosis.

“The ultimate goal is to find reliable therapies to overcome acquired resistance in melanoma patients, improving their prognosis.”

**How would you explain the main findings of your paper to non-scientific family and friends?**

In my paper, I developed a system that allows the study of the impact of a given gene on melanoma incidence in zebrafish and to do so at the desired time during fish life. I employed this innovative system to demonstrate that increased levels of a short RNA molecule called miR-204 result in increased melanoma incidence. My system is very versatile and I hope that the zebrafish community will use it to study other candidate genes involved in melanoma formation and progression.

**What are the potential implications of these results for your field of research?**

My paper can be described as a technical report with biological implications. In the context of the well-established BRAFV600E; p53-/- melanoma model in zebrafish, we developed an inducible system, which is based on Tet-ON technology and allowed us to overexpress and inhibit miR-204 in a spatio-temporal controlled way. The system is pretty straightforward, it is not leaky and the modulation of target gene expression that it induces is robust. Therefore, being based on Gateway technology (a modular cloning technique based on interchangeable vectors), we propose it as a versatile tool for the study of the impact of different classes of genes (both coding and non-coding) in different types of diseases.

“My paper can be described as a technical report with biological implications.”

**What has surprised you the most while conducting your research?**

This is the first published work performed on zebrafish by the Poliseno lab. When I joined the lab, I had never worked with zebrafish before and we had just acquired the BRAFV600E; p53-/- melanoma model. Yet, we managed to create a new inducible system, which, potentially, can be useful for the entire zebrafish community. I consider this my greatest scientific accomplishment, so far!

**What, in your opinion, are some of the greatest achievements in your field and how has this influenced your research?**

In the last years, the use of zebrafish as a cancer model has been introduced, setting aside the idea of zebrafish as just a developmental model. Tumors that develop in zebrafish resemble in fact human tumors in terms of histological features and expression profile. In particular, my paper relies on the BRAFV600E; p53-/- model, which is one of the most widely used to study melanoma in zebrafish. My project has been influenced by the microRNA field as well. microRNAs are non-coding RNAs that play a central role in cell processes by negatively regulating gene expression. microRNAs are dysregulated in a variety of diseases. Specifically, in cancer they act as tumor suppressors or oncogenes, depending on the role of their targets. Building on cancer modeling in zebrafish and on non-coding RNAs, my paper shows that the microRNA miR-204 has an impact on tumor incidence in the BRAFV600E; p53-/- melanoma model.

**Figure:**
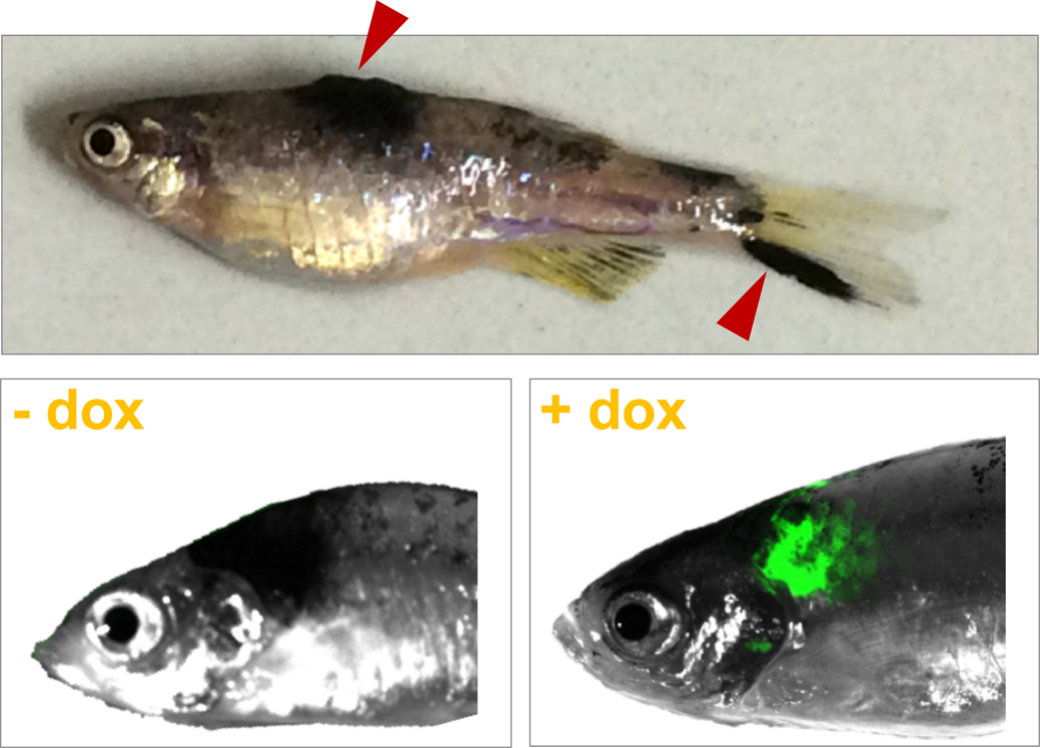
**Representative melanoma tumors that develop in adult fish injected with miniCoopR-inducible vector.**

**What changes do you think could improve the professional lives of early-career scientists?**

In general terms, I think that higher visibility and, at the same time, transparency are necessary for the growth of science itself. Early-career scientists can certainly benefit from increased financial support, objectivity and equality. A good mentor is also necessary to support and guide young researchers in their path toward independence.

**What's next for you?**

After my PhD defence, I was a postdoc at Poliseno's lab for 2 more years. Recently, I became a postdoc in Samuel Sidi's laboratory at Mount Sinai (NY, USA). This is a new opportunity to increase my knowledge both in terms of literature and techniques and to grow as a scientist. After all, in science you never stop learning!
